# Electrocardiographic changes predate Parkinson’s disease onset

**DOI:** 10.1038/s41598-020-68241-6

**Published:** 2020-07-09

**Authors:** Oguz Akbilgic, Rishikesan Kamaleswaran, Akram Mohammed, G. Webster Ross, Kamal Masaki, Helen Petrovitch, Caroline M. Tanner, Robert L. Davis, Samuel M. Goldman

**Affiliations:** 1 Deparment of Health Informatics and Data Science, Parkinson School of Health Informatics and Public Health, Loyola University Chicago, Maywood, IL USA; 2 Department of Biomedical Informatics, Emory University, Atlanta, GA USA; 3 University of Tennessee Health Science Center – Oak Ridge National Laboratory Center for Biomedical Informatics, Memphis, TN USA; 4 Veterans Affairs Pacific Islands Health Care Systems, Honolulu, HI USA; 5 John A Burns School of Medicine, University of Hawaii, Honolulu, HI USA; 6 Pacific Health Research and Education Institute, Honolulu, HI USA; 7 Department of Neurology, University of California-San Francisco, San Francisco, CA USA; 8 Parkinson’s Disease Research Education and Clinical Center (PADRECC), San Francisco Veterans Affairs Health Care System, San Francisco, CA USA; 9 Division of Occupational and Environmental Medicine, University of California-San Francisco, San Francisco, CA USA; 10 San Francisco Veterans Affairs Health Care System, San Francisco, CA USA; 11 Loyola University Chicago, 2160 S First Avenue, CTRE, Bldg. 115, Room 127, Maywood, IL 60153 USA

**Keywords:** Parkinson's disease, Statistics, Diagnostic markers, Predictive markers

## Abstract

Autonomic nervous system involvement precedes the motor features of Parkinson’s disease (PD). Our goal was to develop a proof-of-concept model for identifying subjects at high risk of developing PD by analysis of cardiac electrical activity. We used standard 10-s electrocardiogram (ECG) recordings of 60 subjects from the Honolulu Asia Aging Study including 10 with prevalent PD, 25 with prodromal PD, and 25 controls who never developed PD. Various methods were implemented to extract features from ECGs including simple heart rate variability (HRV) metrics, commonly used signal processing methods, and a Probabilistic Symbolic Pattern Recognition (PSPR) method. Extracted features were analyzed via stepwise logistic regression to distinguish between prodromal cases and controls. Stepwise logistic regression selected four features from PSPR as predictors of PD. The final regression model built on the entire dataset provided an area under receiver operating characteristics curve (AUC) with 95% confidence interval of 0.90 [0.80, 0.99]. The five-fold cross-validation process produced an average AUC of 0.835 [0.831, 0.839]. We conclude that cardiac electrical activity provides important information about the likelihood of future PD not captured by classical HRV metrics. Machine learning applied to ECGs may help identify subjects at high risk of having prodromal PD.

## Introduction

Parkinson’s disease (PD) is a progressive disabling neurodegenerative disorder affecting approximately one million Americans and 50,000 new cases are diagnosed annually^1^. By the time PD becomes clinically apparent, there is more than 50% loss of substantia nigra neurons and an 80% decline in striatal dopamine levels^2,3^. The disease process may be active years or even decades before classical motor features are apparent^3^. Diagnostic tools to identify early prodromal features are essential in order to develop and initiate putative therapeutic agents to slow disease progression.

PD is increasingly recognized to be a systemic disorder with widespread anatomic involvement and nonmotor symptoms including early autonomic pathology and cardiac sympathetic denervation^1^. PD pathology affects the reflex cardiovascular control systems, manifesting as reduced beat-to-beat heart rate variability (HRV) in patients with prevalent disease^4^. Such an effect can be shown noninvasively in prevalent PD subjects using HRV metrics derived from 5-min electrocardiogram (ECG) tracings. Although a prospective study showed that low HRV determined from a 2-min ECG is associated with 2–threefold higher risk for PD^5^, the value of the ECG in predicting prodromal disease has not been established. This may be because heart rate is a function of distance between two R peaks and it does not fully capture all the information reflected within electrocardiograms. A more sophisticated way of modeling electrical activity of the heart may help in identifying prodromal disease.

In this manuscript, we hypothesized that early autonomic features of PD are detectable using machine learning, and tested this hypothesis using standard 10-s ECGs collected from participants in the prospective Honolulu-Asia Aging Study (HAAS).

## Results

### Cohort characteristics

All participants were Japanese American males with characteristics described in Table 1. The age at time of ECG followed a normal distribution for all three subject groups: controls (Kolmogorov–Smirnov Test (KS) p = 0.44), prodromal PD (KS p = 0.14) and prevalent PD (KS p = 0.69). There were no significant differences in mean age at the time of ECG between those with prevalent PD, prodromal PD or controls (ANOVA, p = 0.35). Among those with prodromal PD, the mean duration from ECG until PD diagnosis was 4.3 years (Standard Deviation (SD) 2.4). Among prevalent cases, ECGs were recorded on average 5.4 years (SD 2.5) after first diagnosis of PD. In our cohort, 6 of 25 controls, 5 of prodromal PD cases, and 1 of 10 prevalent PD cases had diabetes.

**Table 1:** Subject characteristics.

	Control (n = 25)	Prodromal PD (n = 25)	Prevalent PD (n = 10)
Age at ECG Mean (SD), range	78.0 (3.7), 72–88	77.6 (4.9), 72–88	79.9 (4.0), 72–85
Age at PD diagnosis Mean (SD), range	–	81.9 (4.8), 74–91	74.5 (5.3), 62–80
Years from ECG until PD Mean (SD), range	–	4.3 (2.4), 1–8	− 5.4 (2.5), − 2 to − 10
Years follow up in controls until death (all controls are deceased) Mean (SD), range	12.3 (4.6), 5–20	–	–
Had autopsy	10/25 (40%)	6/25 (24%)	5/10 (50%)

### Heart rate variability metrics

For each ECG, we calculated nine HR characteristics; mean, median, standard deviation, kurtosis, skewness, minimum, maximum, range, and coefficient of variation. Table 2 summarizes these HR characteristics for prodromal PD, controls, and prevalent PD cases.

**Table 2:** Mean HR characteristics with 95% confidence intervals.

HR characteristics	Controls (n = 25)	Prodromal PD (n = 25)	Prevalent (n = 10)
Mean	65.30 [61.93, 68.68]	64.50 [59.99, 69.02]	68.24 [61.72, 74.76]
Median	65.16 [61.75, 68.58]	64.21 [59.39, 69.03]	68.30 [61.75, 74.85]
Standard deviation	2.65 [1.49, 3.80]	3.87 [1.07, 6.67]	1.20 [0.42, 1.98]
Kurtosis	3.20 [2.26, 4.13]	2.54 [2.21, 2.87]	2.48 [2.01, 2.95]
Skewness	0.19 [− 0.25, 0.63]	0.10 [− 0.17, 0.36]	0.02 [− 0.36, 0.40]
Maximum	69.83 [64.81, 74.86	71.57 [63.56, 79.57]	70.21 [63.32, 77.10]
Minimum	61.00 [57.65, 64.34]	58.90 [54.32, 63.49]	66.21 [59.81, 72.83]
Range	8.84 [4.48, 13.19]	12.66 [3.53, 21.79]	3.90 [1.28, 6.51]
Coefficient of variation	3.85 [2.37, 5.34]	5.58 [1.75, 9.41]	1.74 [0.62, 2.86]

Only Skewness (KS p > 0.05) among nine HR variables (KS p > 0.05) followed a normal distribution. There were no significant differences in means of Skewness between three groups (ANOVA p = 0.86). Among other eight HR variables, there was no variable significantly differed between three groups (Kruskal–Wallis Test p > 0.05).

### Signal processing features

The feature selection step revealed 25 features significantly different for prodromal cases and controls (Mann–Whitney-U test, p < 0.05). Of those features, 19 were related to Fast Fourier Transform, while 2 were related to signal complexity, and included features derived from continuous wavelet transform with various parameters. Some signal energy and quantile mass of time series features were also significantly different for two groups (Mann–Whitney-U test, p < 0.05). These features were then analyzed using Logistic Regression. However, the results of the binary classification did not yield favorable results and therefore we did not pursue these features any further. Using 25 signal processing features and PSPR, the model yielded an average fivefold cross-validation sensitivity and specificity of 0.62 and 0.61.

### PSPR features

**Figure 1:**
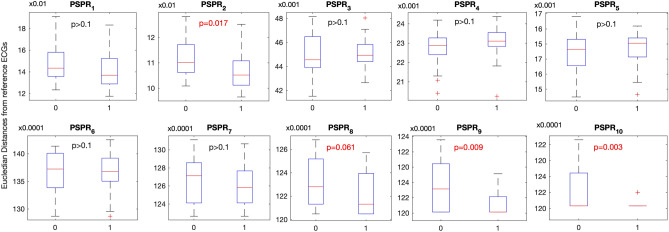
Comparison of prodromal PD (**1**) and control ECGs (**0**) based on PSPR features. Note that PSPR features represent how a given ECG (from prodromal PD subjects or control) differs (dissimilarity or distant) from the ECGs of subjects with prevalent PD. This implies that the dissimilarity between ECGs of prodromal PD and prevalent PD are smaller (more similar) than the dissimilarity between controls and prevalent PD ECGs (less similar).

Figure 1 summarizes the values of 10 PSPR features calculated for 25 Prodromal PD subjects and 25 Controls. None of the 10 PSPR features followed a normal distribution (KS p < 0.01). Among ten PSPR features, three differed significantly between controls and prodromal PD cases (Mann–Whitney U test, p < 0.05).

### Model building to distinguish between prodromal PD ECGs and control ECGs

We built logistic regression models with backward elimination using 10 PSPR features and 9 h characteristics to distinguish between 25 Prodromal PD and 25 Control ECGs. The final model selected four PSPR features (PSPR for pattern lengths of 2, 7, 8, and 9) as predictors of PD and yielded an AUC with 95% CI of 0.90 [0.80, 0.99].

The logistic regression model obtained using all 50 ECGs provides a sensitivity of 84.00% and specificity of 80.00% when a cut off value of 0.5 was used to convert predicted probabilities into binary class predictions. Note that we did not include age or other comorbid conditions in the model, since our goal was to investigate the predictive value of ECG features and because there was no significant difference between the age of cases and controls (p < 0.05; both ANOVA and Mann–Whitney U test).

**Figure 2:**
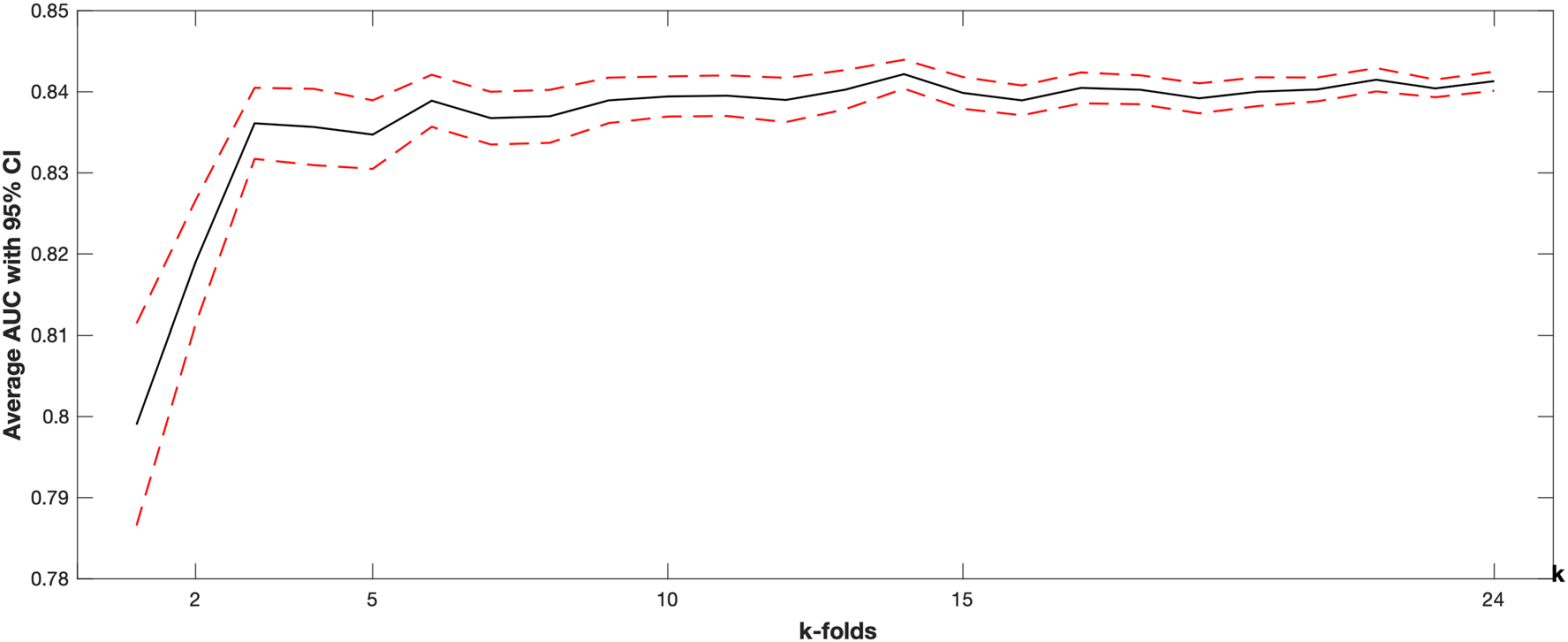
k-folds cross-validation results. The solid black line represents the average cross-validation result, while the dashed red line is the corresponding 95% confidence interval. Increasing ‘k’ indicates a larger training and smaller testing set. For example, when k = 2, a model is trained on 1 × (50/2) = 25 subjects and tested on the remaining 25. When k = 10, a model is trained on 9x(50/10) = 45 and tested on the remaining 5.

We also implemented a cross-validated logistic regression models to show whether extracted ECG features may provide generalizable results or not. Figure 2 summarizes the k-fold cross-validation results in terms of average AUC with 95% CI obtained at different ‘k’ values of k-fold.

## Discussion

Early identification of prodromal PD is an essential step as we progress toward implementing disease modifying therapeutic interventions. The current work took advantage of prospectively collected ECGs to develop predictive models to distinguish between control and prodromal PD subjects. Traditional heart rate variability metrics showed no significant difference between controls and subjects. 25 various signal processing features among 794 features were selected using a univariate statistical approach, but their individual classification performance was poor, possibly due to the small sample size.

Three of ten PSPR features measuring dissimilarity to prevalent PD subjects were statistically significantly smaller for prodromal PD compared to controls, suggesting that there are lower dissimilarities (or high similarities) between the prodromal and prevalent PD groups in terms of how the electrical activity of the heart evolves from the beginning to the end of a given 10-s ECG. Specifically, these three PSPR features correspond to two, eight and nine symbol long patterns where each symbol represent 125 ms long section of ECGs down sampled at 8 Hz. In another words, 250 ms, 1,075 ms and 1,250 ms long subsections of ECGs showed significantly different patterns between controls and prodromal PD subjects.

Finally, the stepwise logistic regression model using these 10 PSPR features provided a high classification performance. Furthermore, a cross-validation study confirmed that the results may be generalizable to a cohort with similar characteristics. We note that claiming a broader generalizability require further external validation on a more diverse cohort. Moreover, there are other classification models that are suitable for analysis of raw ECG signals such as convolutional neural networks (CNN). However, as a deep learning methodology, CNN requires a large sample size, therefore, was not implemented in this study.

Lewy pathology is found throughout the autonomic nervous system in PD^6^. The dorsal motor nucleus of the vagus nerve is thought to be among the earliest affected structures in disease evolution^7^, and pathology in sympathetic and parasympathetic ganglia and cardiac nerves and associated cardiac de-afferentation are consistently seen in early PD^8–11^. For this reason, cardiac sympathetic de-afferentation as measured by metaiodobenzylguanidine^6,7,12^ (I-MIBG) scintigraphy serves as a supportive criterion for the clinical diagnosis of PD in the MDS-PD diagnostic criteria^13^. Cardiac autonomic pathology and de-afferentation are also seen in association with incidental nigral Lewy bodies at post-mortem (ILB)^10^, and as early as 2007 it was proposed that neurocardiologic testing might provide a biomarker for prodromal disease^14^. However, MIBG scintigraphy is invasive and expensive, and is not a viable tool for population-level screening. Thus, the present work investigated whether the ubiquitous, standard 10-s 12-lead EKG might serve as a useful biomarker for prodromal PD.

Berg et al.^13^ proposed a classification model that combines predictors of prodromal PD (REM sleep behavior disorder, olfactory impairment, hyperechogenicity of substansia nigra) with epidemiologic risk factors for PD (sex, occupational exposure to pesticides or solvents, caffeine use, smoking, family history of PD). Our results suggest that early pathologic involvement of cardiac autonomic innervation might be detectable using standard 10-s ECGs in concert with machine learning tools. However, despite the supportive cross validation implemented here, this work requires external validation in other cohorts.

Our study has some major limitations. Although our cross-validated results are promising, the sample size of 60 is very small and could be confounded by a variety of factors. Furthermore, our cohort only included men of Japanese-American descent. Future work will focus on validation of our results in larger and more diverse cohorts. Additionally, subjects with major cardiovascular diseases or those taking medications potentially affecting ECGs were excluded. The impact of these and other common comorbidities and medications on model performance requires further investigation in a larger cohort.

We conclude that the electrical activity of the heart carries important information about the onset of PD that can be detected with a standard 10-s ECG, but that classical heart rate variability metrics are relatively insensitive to early PD pathology. It is possible to capture additional informative data by sophisticated analysis of ECG recordings, and thereby identify subjects at high risk of developing PD. This work suggests that a standard 10-s ECG may serve as a universally accessible, non-invasive, and inexpensive biomarker of prodromal PD. Fast growing technological improvements around wearable devices with ECG tracing functionality may facilitate a broad implementation of such screening algorithms among high risk patients.

## Methods

### Study subjects: Honolulu-Asia aging study (HAAS)

The Honolulu Heart Program prospective cohort study of cardiovascular disease started in 1965 with enrollment of 8,006 Japanese American men born between 1900 and 1919 and living on the island of Oahu^15^. In 1991, HAAS was launched, shifting the focus towards neurodegenerative diseases of aging including PD. Environmental, lifestyle, and physical characteristics including features associated with prodromal PD, were ascertained at baseline and at regular follow-up examinations over 50 years^3^. The institutional review boards of Kuakini Medical Center and the Honolulu Veterans Affairs clinic reviewed and approved the study and written informed consent was obtained from all participants. In addition, a sizeable proportion of participants have undergone post-mortem evaluations for PD-related neuropathology. For the current study, we included 60 individuals with technically good quality ECGs able to be accurately digitized, without arrhythmia or frank conduction abnormality (e.g., bundle branch block), with no history or evidence of myocardial infarction, and not taking beta-blockers or digoxin. The cohort was comprised of 10 subjects who had PD diagnosed prior to ECG recording (‘prevalent cases’), 25 subjects without PD at time of ECG recording, but who developed PD within 1–5 years (‘prodromal cases’), and 25 subjects without PD either at baseline or throughout follow-up (‘controls’). Control subjects were free of CNS Lewy pathology, if neuropathology was available. This research was approved by Loyola University Chicago Institutional Review Board (LU IRB number 212399) with exempt status. Despite our manuscript is a secondary analysis of an existing database, HAAS, the original data collection was carried out by Kuakini Health Systems and was approved by Kuakini Medical Center Institutional Review Board. All methods were carried out in accordance with relevant guidelines and regulations.

### ECG data

Standard 12-lead 10-s resting ECGs were obtained during evaluations conducted from 1991–1993. Paper ECGs were scanned as tiff files at 300 dpi. All ECGs were visually inspected for print quality, arrhythmia, or other significant aberrancies (e.g., recording noise, marked bundle branch block). One well-defined lead was selected for digitization using AMPS ECGscan 3.0^16^.

### Feature extraction

R peaks on the digital ECG recordings were identified and used to calculate heart rate (HR) characteristics (mean, median, standard deviation (SDNN), kurtosis, skewness, min, max, range, and coefficient of variation). Signal processing approaches including Fast Fourier Transform (FFT), signal complexity, and approximate entropy methods with different parameter settings were used. We also extracted features representing changes in ECG recordings using a novel method called Probabilistic Symbolic Pattern Recognition (PSPR)^17–21^, as described below.

### Signal processing features

We utilized the TSFresh Python library^22^, which included unique signal processing methods and their parameters, to extract 794 features from each of the ECG digital signals (control and prodromal group). Each of these features was used to further compare control and prodromal PD subjects using the Mann–Whitney U test, with significance defined at p < 0.05. To minimize potential errors from the converted digital signals, the same digital signals were validated from the ECG image data separately by two authors (AM and RK).

### Probabilistic symbolic pattern recognition (PSPR)

PSPR is a method to process sequential symbolic data in order to understand how a given single sequential data series evolves, and to compare multiple sequential data series regarding their behavior in time. To do that, PSPR drives a probabilistic model, or pattern transition behavior, of each sequential data series and then implements binary comparisons to calculate the Euclidian distance between these probabilistic models. When three series are compared to each other, two series with lower distance have more common behavior compared to two series providing higher distance^17^. When PSPR is applied to real number numeric valued data, such as raw ECG data, each number is first represented with a symbol from a given alphabet with preset length. This discretization can be done either by using arbitrary thresholds or by utilizing domain knowledge. In order to use PSPR for feature extraction from a given data series, data from each series are compared against a set of reference data series. The determination of the reference series is problem specific. In this study, we used 10 prevalent PD subjects as reference data to compare data from 25 controls and 25 prodromal PD subjects.

Our previous analysis showed that PSPR performs best at 8 Hz ECG sampling frequency in problems such as detecting congestive heart failure^18^, cardiac rhythm classification^20,21,23^, atrial fibrillation prediction^24^, and physiologic data analysis^25^. Considering the proven PSPR performance at low sampling frequencies, we down sampled the original ECG signals from 500 to 8 Hz and ran PSPR for all parameter scenarios described in the Methods section. At each run, the PSPR method provided ${n}_{p}$ (max pattern length to model) features. We used these features to build a logistic regression model and calculated the (area under receiver operating characteristics curve) (AUC). The AUC was maximized for the parameter combination of ${n}_{s}=9$ (number of symbols, or the alphabet length), ${n}_{p}=10$. We conducted the rest of the analysis using 10 PSPR features extracted for this parameter setup at 8 Hz.

### Statistical analysis

We tested whether continuous variables were normally distributed using the Kolmogorov–Smirnov test. For normally distributed variables, we used analysis of variance (ANOVA) to test for differences between two or more categories. For non-normally distributed variables, we used the Mann–Whitney U test for two categories, or the Kruskal–Wallis test for more than two categories. Two-tailed p-values < 0.05 were considered significant.

PSPR-generated features were compared between groups using nonparametric tests and then analyzed within logistic regression. We extracted ECG features for controls and prodromal PD cases as described above and used them in a stepwise logistic regression model with backward elimination to distinguish prodromal PD from controls. To account for the small sample size and avoid overfitting, we implemented multiple k-fold cross-validation runs. Number of fold (k) was systematically increased from 2 to 24; for each k, we randomly split data into k folds, built a stepwise logistic regression model using k-onefold of data, and tested the model on the remaining fold. Repeating this process for k times resulted in all predictions being obtained from out-of-sample data. Using these predictions, we calculated AUC. This process was repeated 100 times for each k, with the final results summarized for each k as mean AUC, with a 95% confidence interval.
